# Dr. William Nisbet

**Published:** 1909-04

**Authors:** 


Dr. William Nisbet, of Avon, died at his home in that village February 26, 1909, aged 60 years. He had been ill for about a year and a half with heart disease.

William Nisbet was born at Dalbeattie, Scotland, January 22, 1826. He received his early education there and at Edinburg Medical College. He then spent five years in Europe in study and research, taking his degree from University of Goetengen, Germany.

In 1850 he came to Livingston County settling at Avon, where he has lived since. When he began practising in Avon, he was the only regular physician within a radius of 25 miles. He built up a large and successful practice, conducting a drug store also.

He was a charter member of the Medical Society of the County of Livingston and for many years the leading physician in that section of the country, patients coming to him for twenty miles around.

In December, 1855, he married Miss Eliza Nowlen, of Avon, who died in August, 1904. Six children were born to them, three of whom survive him. Charles S. Nisbet of Amsterdam, Nannie Nowlen Nisbet and Harriet Louise Nisbet, both of that village; also one sister, Mrs. Joseph Newall, of Dalbeattie, Scotland.

